# Spatial Equivalent Circuit Model for Simulation of On-Chip Thermoelectric Harvesters

**DOI:** 10.3390/mi11060574

**Published:** 2020-06-06

**Authors:** Simon Lineykin, Moshe Sitbon, Alon Kuperman

**Affiliations:** 1 Department of Mechanical Engineering and Mechatronics, Ariel University of Samaria, Ariel 40700, Israel; 2 Department of Electrical and Electronics Engineering, Ariel University of Samaria, Ariel 40700, Israel; moshesi@ariel.ac.il; 3 School of Electrical and Computer Engineering, Ben-Gurion University of the Negev, Beer-Sheva 8410501, Israel; alonk@bgu.ac.il

**Keywords:** on-chip generator, thermoelectric generator, equivalent circuit model, miniaturized generator, low power generator, thermoelectric harvester, circuit simulator, thermal electrical analogies

## Abstract

Interest in autonomous low-power energy sources has risen with the development and widespread use of devices with very low energy consumption. Interest in thermoelectric harvesters has increased against this background. Thermoelectric harvesters, especially harvesters on-chip, have peculiar properties related to the thermal route, thermal transients, and spatial temperature distribution within the chip. A behavioral model of the harvester is required for engineers to successfully develop voltage converters with maximum power point tracking and energy storage units. There are accurate models based on the finite element method, but these models are usually not compatible with simulators of electrical circuits, and therefore are not convenient for designers. Existing equivalent circuit models fit this requirement, but usually do not consider many parameters. This article proposes an original method that allows simulating spatial thermoelectric processes by analogy with the finite difference method, using electrical circuits simulations software. The study proposes a complete methodology for building the model and examples of simulations of one-, two- and three-dimensional problems, as well as examples of simulation of macro problems in the presence of external thermal and electrical devices, such as heatsink and electrical load.

## 1. Introduction

The thermoelectric properties of materials have been known, and used with greater or lesser intensity, for over a hundred years. Thermoelectric power generators steadily occupy a niche in the market for autonomous power supplies used in space [1,2], in the ocean [3,4,5] and in other hard-to-reach places. Thermoelectric harvesters that turn waste heat into energy have been further developed these days. Such harvesters are increasingly used as an autonomous power source in the nodes of wireless sensor networks. The newest trends in using thermoelectric generators are wearable energy sources for ultra-low-power sensors, harvesters using small temperature gradients as energy sources, and on-chip thermoelectric harvesters [6,7,8,9,10,11,12,13,14,15,16].

There are two main approaches to the analysis and computer simulation of processes taking place in thermoelectric products. The first approach involves the use of finite element methods. This method allows multi-domain simulation with very high accuracy [12,14,17,18]. This method allows simulating an on-chip thermoelectric harvester, considering geometry and technology. At the same time, the method requires large computational resources and is mostly used to calculate a single thermoelectric couple or a small group of thermoelectric couples. The software that performs this type of simulation is usually poorly compatible with electrical circuit simulators, so it is almost impossible to simulate a thermoelectric module together with peripheral devices such as electrical load, converter, etc. Another common way to analyze thermoelectric products is to use a macro-model of the module. The thermoelectric module, in this case, is considered as a certain device in which the laws of energy conservation are satisfied, in which thermal and electrical processes and their interaction take place. The device can exchange energy with the outer world through thermal and electrical terminals [19,20]. Such a model is often performed in the form of an equivalent electrical circuit, in which all thermal processes are presented in the form of equivalent electrical processes [21,22]. This approach is convenient for designers of electrical converters for harvesters, such as maximum power point tracking, etc. Simulations of such a model can be performed using commonly available electrical circuit simulators such as PSPICE [23]. This method, with all its advantages, has proven itself in simulating traditional thermoelectric modules. In the case of planar and especially on-chip planar thermoelectric elements, the accuracy of the method is often insufficient. This is due to the geometry of planar thermocouples, which is more complex than the geometry of the traditional ones. The temperature distribution along with the p- and n-legs of the thermocouple is different. The heat extenders at the warm and the cold edges of the thermoelectric elements are bulky, and their thermal behavior should be considered. The Thomson effect is not negligible in planar thermoelectric devices due to the significant length of the legs.

In this study, we offer a technique that is designed to combine the advantages of both methods. This technique enables modeling and simulation of thermo-electric processes with the spatial equivalent circuit model. This technique allows the performance of joint simulations of thermoelectric devices together with external thermal and electrical elements, such as heat sources, heatsinks, and electronic converters and loads using electrical circuits simulation software. Equivalent R-C circuits have been used before for simplified simulations of thermal and electrical processes [24]. In some records on heat and mass transfer, methods of thermoelectric analogies are proposed for the analysis of both static and dynamic processes [25].

The innovation proposed in this work is the creation of a generic model of elemental volume suitable for different types of materials, including anisotropic materials, as well as materials whose thermal and electrical properties are significantly dependent on temperature. The proposed model complements existing methods of solution of the heat transfer task employing the equivalent circuit of the elemental volume by considering such processes as the Joule heating, as well as the Seebeck, the Peltier, and the Thomson thermoelectric effects.

When using the proposed method, it should be borne in mind that programs for simulating electrical circuits are not designed for an extremely large number of elements. In this sense, finite element methods allowing a tiny mesh are more accurate. Nevertheless, the proposed method allows us to evaluate the thermoelectric system at the level of physical processes without the use of additional resources, to pay attention to some nonobvious phenomena, and to simulate thermoelectric devices operation jointly with electronic devices to demonstrate their mutual functioning.

The article is organized as follows. A brief background on thermoelectric generators and harvesters’ topologies and properties is given in Section 2. The proposed method of the spatial modeling of thermal and thermoelectric processes using equivalent circuit is described in Section 3. Section 4 demonstrates examples of time-domain and steady-state simulation of heat conduction and thermoelectric power generation. Different types of simulations of a thermoelectric element using the proposed model with the same simulation types using the proven finite-difference model simulation are compared in Section 5, and Section 6 summarizes the study, discusses the advantages and disadvantages of the method and directions for future research.

## 2. Background on Thermoelectric Harvesters

**Figure 1:**
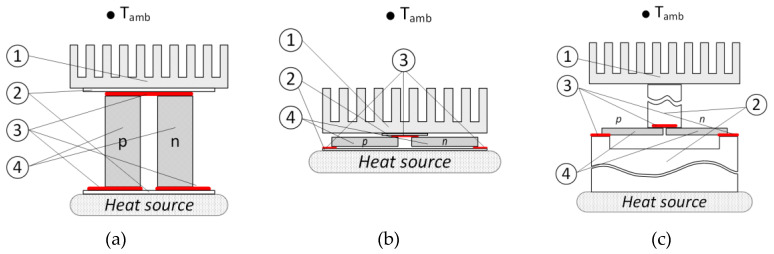
The typical topologies of thermoelectric elements: (**a**) traditional, (**b**) planar, and (**c**) on-chip planar. The common elements of the topologies are: (1) heatsinks, (2) electrically isolating thermal extenders, (3) electrical contact pads, and (4) thermoelectric pairs.

Figure 1 depicts the three main types of thermoelectric elements. All the thermoelectric elements shown in the figure have a similar structure but differ in external dimensions, topology, geometry, and the number of elements in one module. The thermoelectric pair, indicated in the figure by the number 4 and the letters p and n, provides thermoelectric conversion. It is an integral part of the thermoelectric generator, consists of dissimilar materials, usually a pair of semiconductors of the p- and n-type with a pronounced Seebeck coefficient. About 30 of the most popular thermoelectric materials are described in [26]. The most common applications for each of the materials are also reported there. In the current work, materials that have properties close to those of polysilicon and (Bi_0.5_Sb_0.5_)_2_Te_3_ are used in the examples. Each of the “legs” may have a different geometry and may even be composed of segments from several different materials [27]. The thermoelectric “legs” are electrically connected and also connected to the other thermoelectric pairs in the module using conductive contacts, indicated in Figure 1 by the Number 3 and highlighted in red. A thermoelectric pair, together with the contact pads, are enclosed between the elements, Number 2, which provide electrical insulation, good thermal conductivity, and the mechanical rigidity of the entire structure. The thermal gradient over the thermoelectric pair leads to the appearance of electromotive force (EMF). The source of the thermal gradient is the difference between the temperature of the heat source and the ambient temperature T_amb_. In most cases, conductive heat transfer occurs between the thermoelectric module and the heat source, while convective heat transfer takes place between the thermoelectric module and T_amb_. through heatsink at Number 1.

In a traditional thermoelectric element shown in Figure 1a, the size of the thermoelectric pair is dominant. Thus, the electrical and thermal resistances of the pair are dominant too. The electric contact resistance ((3) in Figure 1) and the thermal resistance of the insulating layers ((2) in Figure 1) are often assumed as negligible or less significant compared to the thermal and electrical properties of the pair itself [21,27,28]. A planar thermoelectric element shown in Figure 1b differs from the traditional one in that heat flows along its “legs” orthogonal to the direction of the general temperature gradient. With this arrangement, secondary temperature gradients along the “legs” can occur, which means that the temperature distribution along the “legs” becomes more complicated [29]. A thermoelectric pair in a planar on-chip thermoelectric element, shown in Figure 1c has a micrometric size [8,9,10,17,18]. Millions of thermoelectric pairs can be placed in one module. Such pairs are usually located on a silicon substrate. Special cavities in the substrate serve to thermally isolate the common contact between the p and n “legs”. There may be air or a vacuum inside the cavities. The heat-conducting elements above and below the thermoelectric pair (Number 2 in Figure 1) transmute into thermal extenders, one of them is a silicon substrate and the other is a pillar consisting of metal layers and vias, whose dimensions significantly exceed the dimensions of the thermoelectric pair. This means that thermal processes outside the thermoelectric pair become no less significant than the processes within the pair itself. Besides, thermal gradients within the substrate can lead to different thermal conditions for different pairs. In some cases, this process must also be considered [17]. In addition to this, complementary metal–oxide–semiconductor (CMOS) or micro-electro-mechanical systems (MEMS) technology sometimes does not allow for the use of well-studied materials, requiring the trial of more recently developed ones. The thermal and electrical properties of such new materials, as well as their mutual influence, are still being researched. Many of the existing models of thermoelectric devices that reliably work with traditional thermoelectric elements do not take into account the asymmetry of heat distribution inherent in planar thermoelectric elements, the dependence of the thermal and electrical properties of materials on local temperature, and the inhomogeneity of temperature conditions for thermoelectric elements located at a considerable distance from each other. The spatial 3D model is required to analyze the operation of such thermoelectric modules.

## 3. Modeling

**Figure 2:**
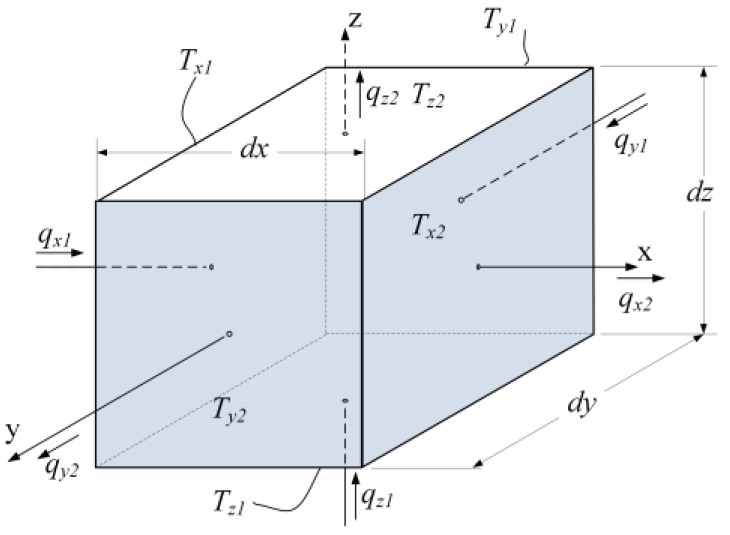
An elemental volume of the solid under analysis.

The spatial numerical solution of heat conduction in solids by the finite difference method includes dividing the body into several elemental volumes, compiling equations of temperature-heat flow at its boundaries and internal energy conversions. This method is described in detail in literature on heat transfer, for example [25]. An example of such an elemental volume is shown in Figure 2. The elemental volume is located in the Cartesian coordinate system. The faces of the elemental volume are named according to their location as *x*_1_, *x*_2_, *y*_1_, *y*_2_, *z*_1_, and *z*_2_ correspondingly. The temperatures of the corresponding faces and the heat fluxes flowing in or out of the elemental volume have corresponding subscripts.

The law of energy conservation allows us to argue that the total heat entering the elemental volume leads to a change in the internal energy of this elemental volume. From this statement, the heat equation is derived,

(1) $$(q_{x1}-q_{x2})+\left(q_{y1}-q_{y2}\right)+(q_{z1}-q_{z2})+q_{g}=\frac{dE}{dt}$$

where $q_{ji}$ is a value of the heat flowing into or out of the elemental volume through the corresponding face, j means the coordinate axis, n means number 1 or 2 of the face, (see Figure 2), $q_{g}$ is the heat generated within the elemental volume, and E is the internal energy of the elemental volume.

The Fourier’s Law of heat conduction along the *x*-axis has the following differential form:

(2) $$q_{x}=-kA_{x}\frac{\partial T}{\partial x}$$

where *k* is thermal conductivity, [$W\cdot m^{-1}\cdot K^{-1}$], $A_{x}$ is the area of the face *x*_1_. Rewriting (2) in the form of differences for corresponding axes, and expressing internal energy of the elemental volume as the specific heat of material $c_{p},\;\left[J\cdot {\mathrm{kg}}^{-1}\cdot {\;K}^{-1}\right]$ times its temperature, we get:

(3) $$k\cdot \frac{\left(\Delta y\cdot \Delta z\right)}{\Delta x}\cdot \Delta T_{x}+k\cdot \frac{\left(\Delta x\cdot \Delta z\right)}{\Delta y}\cdot \Delta T_{y}+k\cdot \frac{\left(\Delta y\cdot \Delta x\right)}{\Delta z}\cdot \Delta T_{z}+q_{g}=c_{p}\cdot m\cdot \frac{\partial T_{C}}{\partial t}$$

where $\Delta T_{j}=T_{j2}-T_{j1}$, and j=x, y, or z. The $T_{C}$ is a temperature of the mass of the elemental volume. For simplicity, it is assumed that the mass of the elemental volume is concentrated in the middle of the volume.

### 3.1. Electrical Analogy

In this article, we will first use the equivalent circuit developed in [24], then expand it to include an electrical domain for mutual simulation of thermal and electrical processes, and finally add thermoelectric process to the expanded circuit. The thermoelectric analogies used in [19,21,24,25] are listed in Table 1.

For the sake of reconstructing the equivalent circuit provided in [24], let us rewrite Equation (3) using the relations listed in Table 1.

(4) $$\frac{\Delta T_{x}}{\Theta_{x}}+\frac{\Delta T_{x}}{\Theta_{x}}+\frac{\Delta T_{x}}{\Theta_{x}}+q_{g}=C_{T}\frac{\partial T_{C}}{\partial t}\;$$

**Figure 3:**
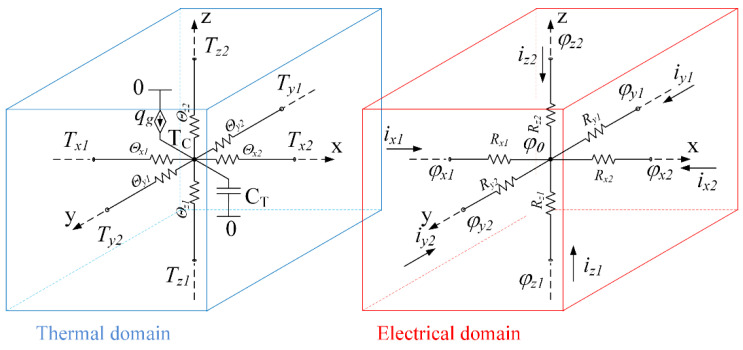
Thermal and electrical domains of the equivalent circuit for mutual simulation of electrical and thermal processes taking place in an elemental volume. The ground of the thermal domain is absolute zero temperature, 0 kelvin. The thermoelectric processes are not included in the model at this stage.

An equivalent circuit corresponding to (4) is shown in Figure 3, thermal domain. A mass of the elemental volume is presented as an equivalent capacitor $C_{T}$. One terminal of the capacitor is connected to the central junction, which is separated from each of the faces by the corresponding thermal resistance where $R_{j1}=R_{j2}=0.5\;R_{j}$, where subscript j means x−, y− or z−axis name. The second terminal of the capacitor is connected to the common point of the thermal domain (0, kelvin). An equivalent current source $q_{g}$ that represents the heat generated within the elemental volume is also connected between zero potential and a central junction. An initial condition (IC) of the equivalent capacitor is the initial temperature of the elemental volume. The boundary conditions may be set by the current source as an equivalent of heat flow, as an equivalent of temperature, by a voltage source, or by contact with neighboring elemental volume.

If the heat *q_g_* generated inside the elemental volume is the result of the Joule heating, then this process can also be included in the model for simulation by adding an electrical domain to the model: see Figure 3, electrical domain. The electrical model includes only six resistors, equivalent to the electrical resistance of the elemental volume in each dimension. $R_{j1}=R_{j2}=\frac{R_{j}}{2}$, see Table 1. The value of the Joule heating is equal to a voltage drop over each resistor ($v_{R_{jn}}$) times current ($i_{jn}$) through this resistor.

(5) $$q_{g}=\underset{\begin{array}{c} j=x,y,z\\ n=1,2 \end{array}}{\sum }v_{R_{jn}}\cdot i_{jn}$$

### 3.2. Extending the Model by Introducing the Thermoelectric Processes

The model shown in Figure 3 should be expanded to consider the Seebeck and Peltier thermoelectric effects. In an expanded form, this model will be suitable for modeling thermoelectric elements.

**Figure 4:**
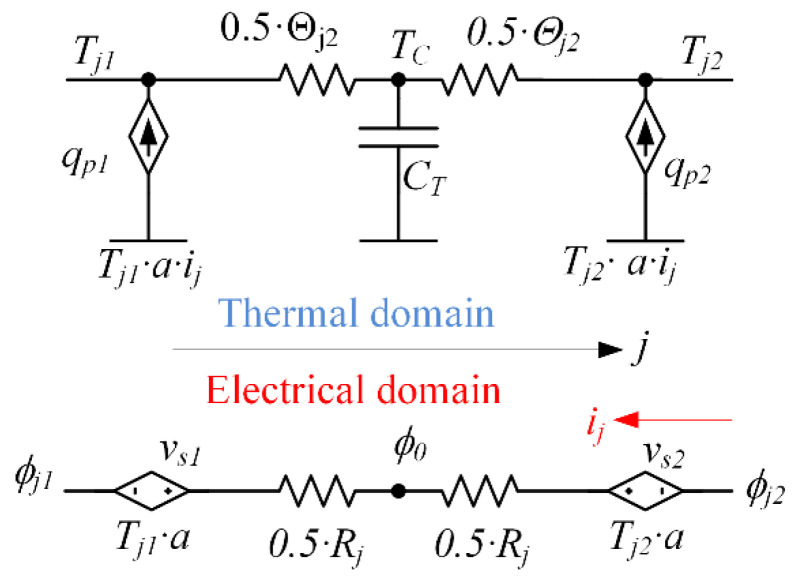
Illustration of thermoelectric effects and interaction between thermal and electric domains in an elemental volume from Figure 3. The global axis j is used instead of *x*-, *y*- or *z*-axes. $R_{j}$ is the ohmic resistance of the elemental volume on *j*-axis and $\Theta_{j}$ is its thermal resistance on j-axis.

The essence of the Seebeck thermoelectric process is that in the presence of a thermal gradient over the conductor, an EMF appears in the conductor. The magnitude of the EMF is proportional to the temperature drop. This phenomenon is explained by the fact that the greater the energy possessed by the free charge carriers in a conductive material, the higher the temperature of their location zone. As a result, charge carriers drift from the warmer zone of the conductive material towards the colder zone due to diffusion. The coefficient that relates the EMF to the temperature gradient is called the Seebeck coefficient ($\alpha ,\;V\cdot K^{-1}$). In some materials, the Seebeck coefficient is significantly higher than in others. The Seebeck coefficient can be both negative in the case when the charge carriers are negative (electrons) and positive if the charge carriers have a positive sign (holes). A thermoelectric pair, such as the one in Figure 1, consists of two dissimilar materials with Seebeck coefficients of different signs (p- and n-type semiconductors). In the presence of the same temperature gradient, such materials generate EMFs with the opposite sign. These EMFs are summed up due to the galvanic contact between the materials, as shown in Figure 4.

Since the Seebeck effect is a property of the material, it can be described for an elemental volume. Assume that a temperature gradient is applied to the elemental volume along the global axis *J*. The temperatures at the boundaries of the volume are $T_{J1}$ and $T_{J2}$. In this case, the EMF arising at the electrical terminals of this volume is equal to:

(6) $$EMF=\phi_{j1}-\phi_{j2}=\alpha \left(T_{j1}-T_{j2}\right)=\alpha T_{j1}-\alpha T_{j2}$$

Thus, we can say that the electric potential $\phi_{j1}$ appears on the face *j*_1_ of the elemental volume under the influence of the temperature $T_{j1}$, and the electric potential $\phi_{j2}$ appears on the opposite face $j_{2}$ under the influence of the temperature $T_{j2}$. A pair of oppositely directed behavioral voltage sources v_s1_ and v_s2_, equal in magnitude to the thermoelectric potentials $\phi_{j1}$ and $\phi_{j2}$, respectively, are shown in the electrical region of Figure 4. If $T_{j1}>T_{j2}$, then the electric potential on $\phi_{j1}$ surface *j*_1_ is higher than $\phi_{j2}$ on surface *j*_2_; thus, EMF is nonzero.

When a load is connected to electrical terminals, an electric current *i_j_* flows through the volume. In the circuit on Figure 4, the electric current passes through the voltage source $v_{s2}$ in a positive direction, and through the source $v_{s2}$ in а negative direction. In other words, a certain amount of electric power equal to the voltage drop over the voltage source $v_{s2}$ multiplied by the current *i_j_* is converted (pumped) from electrical power at the electrical domain into heat in the thermal domain in face *j*_2_. The electrical negative power is raised at voltage source $v_{s1}$ when the current *i_j_* flows through it in the negative direction. This power is pumped from the thermal domain at face *j*_1_ to the electrical domain. The thermoelectric effect, which leads to the absorption of heat on one surface of the material and the emission of heat on the other surface as a result of an electric current in the material, is called the Peltier effect. Formally, the Peltier effect is expressed as follows:

(7) $$q_{pn}=T_{jn}\cdot \alpha \cdot i_{j}=\phi_{jn}\cdot i_{j},$$

where n is Number 1 or 2 of the face of elemental volume along the corresponding axis.

**Figure 5:**
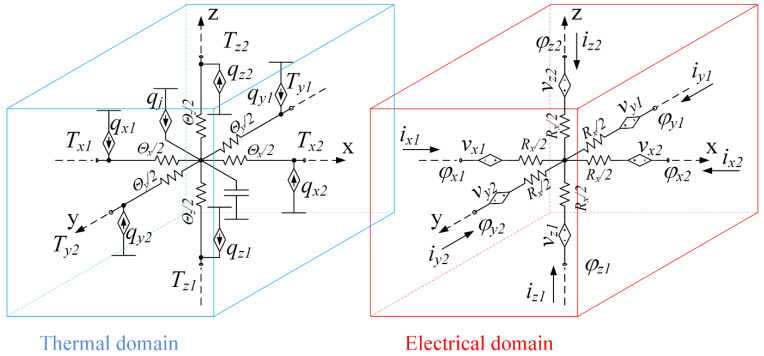
Thermal and electrical domains of the modified equivalent circuit for mutual simulation of electrical, thermal, and thermoelectric processes taking place in an elemental volume.

Using the same approach, to emulate Peltier and Seebeck thermoelectric effects, we can add the corresponding elements into the spatial equivalent circuit shown in Figure 3. Figure 5 depicts a modified spatial equivalent circuit of an elemental volume suitable for simulating thermoelectric processes.

### 3.3. Temperature Coefficient

The two-domain equivalent circuit of the elemental volume of the material shown in Figure 5 already allows simulations of thermoelectric processes in materials. However, this circuit has one significant drawback: it employs physical parameters, such as electrical resistivity, thermal conductivity, and Seebeck coefficient, as constant values. In practice, all of these physical quantities are subject to variations due to temperature. These changes are not negligible. An increase in the electrical resistance of a material with temperature leads to significant losses in output power, and the temperature dependence of the Seebeck coefficient on temperature significantly affects the output voltage of a thermoelectric generator. This phenomenon is known as the Thomson thermoelectric effect. This effect can be neglected in the analysis of a traditional thermoelectric generator, but with planar or on-chip generators, this effect should not be neglected.

As a simplified function of the dependence of a certain parameter *X(T)* on temperature, one can use the first-order function, expressed using the thermal coefficient:

(8) $$X\left(T\right)=X^{*}\cdot \left(1+x\cdot \left(T-T^{*}\right)\right)$$

where $X^{*}$ is a value of parameter *X(T)* measured at reference temperature $T^{*}$, *T* is an actual temperature, and *x* is a temperature coefficient. Thus, we can express the necessary parameters of materials in Form (8)

(9) $$\rho_{\Omega }\left(T\right)=\rho_{\Omega }^{*}\cdot (1+\rho_{\Omega 0}\cdot \left(T-T^{*}\right))$$

(10) $$k\left(T\right)=k^{*}\cdot \left(1+k_{0}\cdot \left(T-T^{*}\right)\right)$$

(11) $$\alpha \left(T\right)=\alpha^{*}\cdot \left(1+\alpha_{0}\cdot \left(T-T^{*}\right)\right)$$

The physical parameters of most of the materials, as well as corresponding thermal coefficients, are available in the literature.

Temperature is one of the variables to be simulated: we want to define a parameter of a model as a function of temperature. The values of the electrical resistors and the thermal resistors represented in the model by the equivalent electrical resistors used in the model are temperature invariable. The temperature dependent resistors can be modeled using behavioral sources.

Ohm’s law states the following: the voltage drop across the resistor is proportional to the current:

(12) $$V_{R}=I_{R}\cdot R\;or\;I_{R}=\frac{V_{R}}{R}$$

Thus, the voltage drop across the resistor due to current can be emulated with a current-controlled voltage source (CCVS). When using a CCVS, the output port of the voltage source is connected to the branch of the circuit instead of the resistor. The voltage drop, in this case, is set to be equal to the product of current flowing through the branch and the resistance value. The resistance value can be specified as a number (temperature independent value) as well as an Equations (9) and (10).

The temperature dependence of the Seebeck coefficient can be introduced into the model by replacing the constant value of the α in the $v_{jn}$ sources with Equation (11).

**Figure 6:**
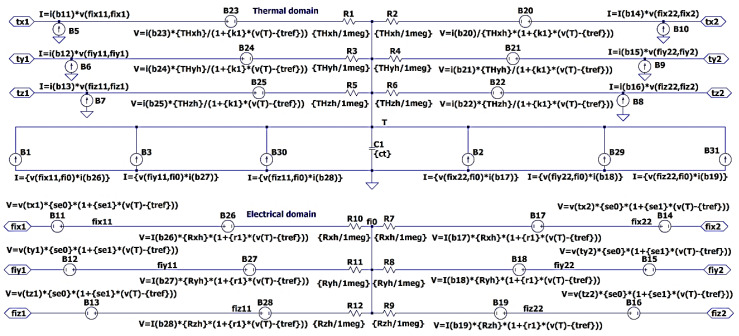
An equivalent circuit for simulation of thermoelectric processes in elemental volume. The equivalent circuit is designed as a hierarchical block to simulate in LTSpice software [30]. Parameters ct, Rxh, Ryh, Rzh, r1, se0, se1, k1, THxh, THyh, THzh, and tref means $C_{T}$, $R_{x}/2$, $R_{y}/2$, $R_{z}/2$, $\rho_{\Omega 0}$, $\alpha^{*}$, $\alpha_{0}$, $k_{0}$, $\Theta_{x}/2$, $\Theta_{y}/2$, $\Theta_{z}/2$, and $T^{*},$ respectively. The resistors $R_{1}-R_{12}$ in the scheme have insignificant values and serve to prevent the problem of convergence during circuit simulation.

An example of a hierarchical block representing the equivalent circuit of an elemental volume from Figure 5 using the described technique is shown in Figure 6. The block has 12 bidirectional connectors named as $fi_{jn}$ for the electrical domain and $t_{jn}$ for the thermal domain. Subscript j is a corresponding x, y, or z axis name, and n is the surface number.

**Figure 7:**
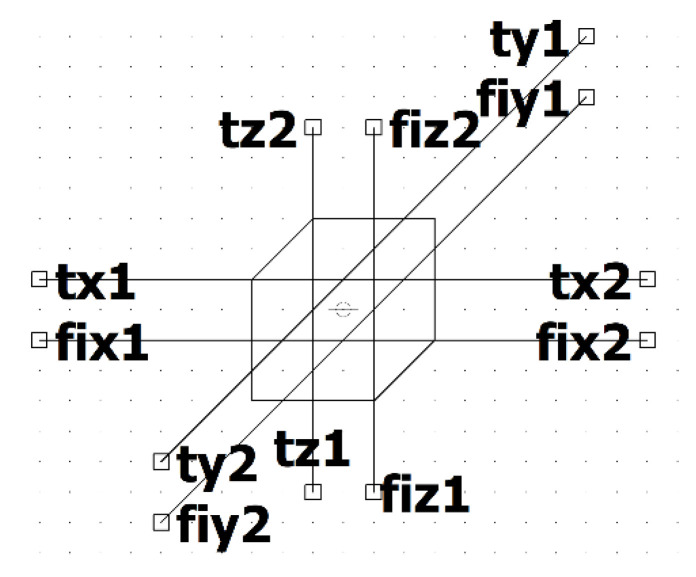
The symbol of the hierarchical block of the 3D equivalent circuit of elemental volume shown in Figure 6 for LTSpice [30].

In Figure 7 an example of the schematic symbol proposed for such a type of equivalent circuit is shown. The symbol of the hierarchical block can be of any shape. We propose to make it look like a cube, in order to emphasize the association of the equivalent circuit with the 3D element of the spatial structure. Each face of the cube has two terminals: one terminal ($fi_{jn}$) belongs to the electrical domain and the second terminal ($t_{jn}$) belongs to the thermal domain.

The electrical domain of one block may be joined to the electrical domain of other blocks or electrical elements such as an electrical load or a current or voltage source. The thermal domain of one block may be connected to the thermal domain of another block, or equivalent electrical elements representing a temperature source, heat sources, or thermal impedances. The thermal and electrical domains should remain isolated from each other. 

## 4. Examples of Simulations

In this part, examples of heat-conducting simulation using the proposed method for solving various spatial problems are demonstrated. These examples relate to solving one-, two-, and three-dimensional heat conduction problems in solids, to simulating thermoelectric processes in a single material and in a thermoelectric pair. Further, this section analyzes the behavior of a group of thermoelectric elements under similar conditions, macro models including millions of thermoelectric pairs located on a single substrate, and peripheral devices such as a radiator and an external electrical load.

The materials used in the examples have physical properties close to those of the materials normally used for building on-chip thermoelectric generators in CMOS technology. Some properties of the materials are deliberately exaggerated in order to visualize certain physical phenomena, such as the Thomson effect. The topology of the sample thermoelectric generator is also demonstrated in a simplified form with approximate geometric dimensions.

### 4.1. Heat Conduction Simulation 

**Figure 8:**
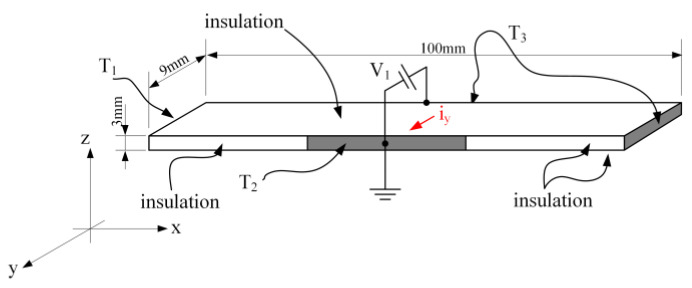
An example of the problem of heat conduction in a solid with partial thermal insulation, initial and boundary conditions, and internal heat generation.

As the first example, consider the heat conduction problem shown in Figure 8. The plate is made of a material whose properties are close to those of aluminum. The plate is thermally insulated from above, from below, and partially from the front (insulation is shown in white in the picture). Prior to the simulation, the plate had an initial temperature equal to the ambient temperature T_amb_. At the beginning of the simulation, boundary conditions were applied to the plate in the form of temperatures T_1_ on the left face, temperature T_2_ in the middle third of the front face, and temperature T_3_ on the right and back faces. At the beginning of the experiment, the front and back faces of the plate were electrically grounded. The electrical potential V_1_ was supplied to the rear face of the plate with a delay t_1_ relative to the start of the experiment.

The parameters used in the simulation are collected in Table 2.

**Figure 9:**
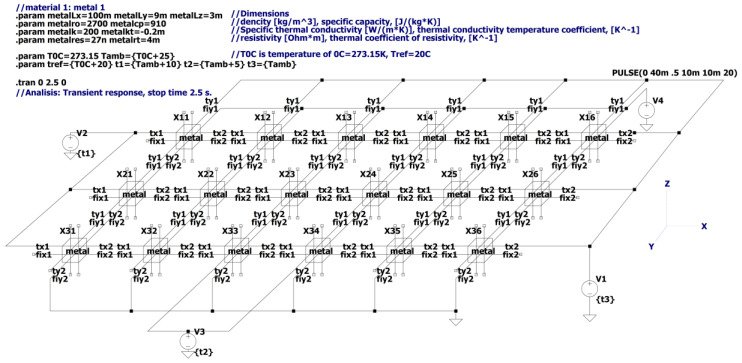
The electrical equivalent circuit in LTSpice schematic editor for the heat conduction problem from Figure 8. The circuit includes 18 blocks shown in Figure 6. The blocks are designated as $X_{ij}$, where *i* is the row number and the j is a block’s number in the row.

Figure 9 shows the electrical equivalent circuit of the problem depicted in Figure 8, performed in the schematic editor of the LTSpice circuits simulator software. The plate is divided into a mesh of 18 blocks, 6 along the *x*-axis and 3 along the *y*-axis, and one along the *z*-axis as shown in Figure 6. Parameters are listed out on the working page of the circuit editor. Temperatures are given as equivalent voltage sources.

**Figure 10:**
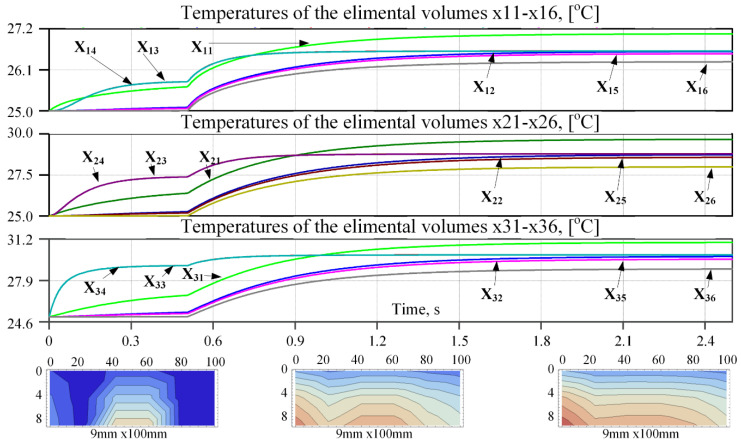
Equivalent voltages representing the temperatures of the central points of each of the 18 elemental volumes. The 1 V of equivalent voltage corresponds to 1 °C. The three plot-panes correspond to three rows of the mesh. The temperature gradient plots correspond to 0.3 s, 1.2 s, and 2.3 s are shown below the time response curves.

The temperature response of each elemental volume of the plate to the boundary conditions and the internal heat generated due to electrical current that turns on after 0.5 s delay is shown in Figure 10 in the form of curves of the equivalent voltage vs. time. The 1 V of the equivalent voltage corresponds to 1 °C.

### 4.2. Thermoelectric Couple

**Figure 11:**
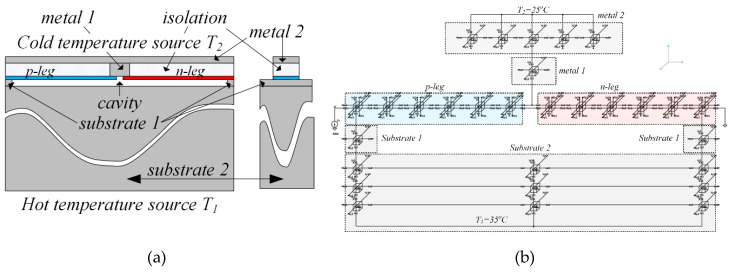
The planar on-chip thermoelectric couple: (**a**) schematic front and side views and (**b**) simulation scheme, built from hierarchical blocks shown in Figure 6 and Figure 7 for different materials.

This section discusses an example of simulating the behavior of a planar on-chip thermoelectric couple Figure 11. Approximate dimensions have been selected based on [7,8,17,23,31]. The thermoelectric couple consists of a pair of semiconductor materials of p- and n-types (*p-leg* and *n-leg* in the figure). The couple is mounted on a silicon substrate with the cavity etched under the cold junction for thermal isolation between the cold and the hot edges of the thermoelectric legs. The hot external edges of the thermoelectric pair have thermal contact with the substrate (*substrate 1* and *substrate 2*) and the substrate, in turn, is connected to a hot temperature source (*T*_1_) at the bottom side of the construction. The cold internal edges of the thermoelectric legs are connected thermally to an upper metal layer (*metal 2*) through the heat-conducting extender made from metal layers and metallized vias. The extender is assumed for the sake of simplicity as a uniform material (*metal 1*). The upper metal layer is connected thermally to a cold temperature source (*T*_2_).

The materials used to model the thermoelectric pair are taken hypothetically because the model is generic. The properties of the materials are close to those of real materials, but we can vary them slightly to obtain more visual simulation results. For simplicity, suppose that there is a vacuum in the cavities and the thermal insulation is ideal. Otherwise, the properties of the insulating material should be added, as shown in [17].

The properties of the materials used in the simulation are tabulated in Table 3.

**Figure 12:**
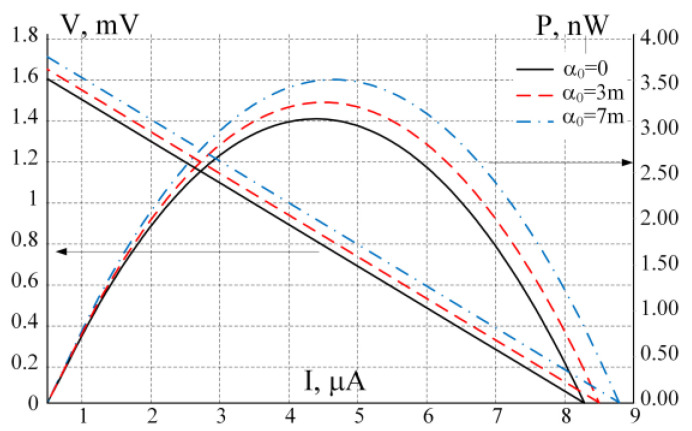
V-I and P-I output characteristics of a single couple shown in Figure 11 for various values of thermal coefficient of Seebeck constants for p- and n-types of thermoelectric materials.

Figure 12 shows the load curves (V-I and P-I) for the single thermoelectric pair being tested. The result was obtained using DC-sweep analysis for various values of the thermal coefficient $\alpha_{0}$ of the Seebeck coefficient α for both p- and n-materials. This experiment demonstrates the Thomson effect on the output characteristics of the module. We remind that $\alpha_{0}$ values are taken to demonstrate the effect only and are not physical properties of specific materials.

### 4.3. Sub-Units’ Simulation

Figure 12 shows the voltage-current and power-current characteristic curves of the single thermoelectric pair, shown in Figure 11. The power yield of one pair is small, but the idea of a thermoelectric harvester on a chip involves the use of many (up to several million) such pairs. All the pairs are normally divided into sub-units of several thousand pairs each, electrically connected in series. The groups can be interconnected in series or parallel, depending on the power requirements, [23]. In [17] it is demonstrated that thermoelectric pairs located at a large physical distance from each other are in slightly different temperature conditions. Nevertheless, the temperature conditions for pairs in close proximity to one another can be considered identical.

As an example of the proposed method’s usage for multiple pairs and sub-units, we analyze the operation of a thermoelectric harvester on a chip that includes 9 sub-units, one thousand pairs each. The elements of one sub-unit are considered to be located close enough to each other so that it can be said that their functioning is identical. In this case, there is no need to multiply the number of blocks. The model of a single pair simulates the behavior of all the pairs in the entire sub-unit.

**Figure 13:**
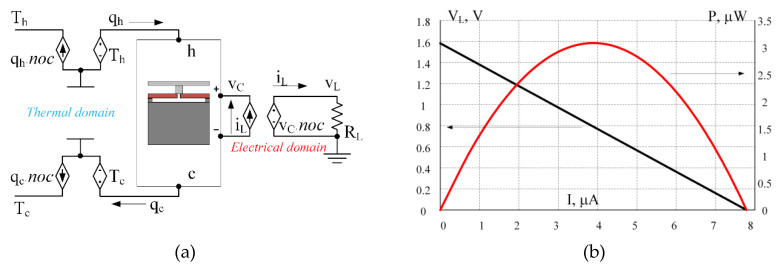
Simulation of a 1k-couples sub-unit using the model of a single couple shown in Figure 11. (**a**) Usage of behavioral voltage and current sources pairs to imitate the series connection of the couples in the electrical domain and parallel connection of the couples in the thermal domain. (**b**) The load curves (V-I and P-I) of the sub-unit with 1k couples.

Electrically, all couples in a sub-unit are connected in series. This means that their total output voltage is equal to the output voltage of a single couple times the number of couples (noc) in the sub-unit. The common electric current of all couples of the sub-unit is the same for all pairs and depends on the load. The electrical domain in Figure 13a demonstrates the implementation of these principles using a pair of behavioral sources of current and voltage. A controlled current source provides an output current of the couple equal to the load current and a controlled voltage source provides the load voltage equal to the voltage of a single couple times the number of couples (noc) of the sub-unit.

The parallel connection of the couples in a thermal domain can be imitated similarly, using two pairs of interdependent sources, see Figure 13a. In this case, it should be remembered that the amount of heat flowing into the couple from the hot side is not equal to the amount of heat flowing from the cold side, since part of the heat is converted to electricity and part spent on heat loss. Thus, the thermal terminals h and c should be considered independent ports, where the common terminal is 0 kelvin. For each of these thermal ports, the corresponding behavioral voltage source sets the equivalent temperature equal to the temperature of the source, and the behavioral current source multiplies the equivalent heat flux of the temperature source by the number of couples (NOC). The more pairs working in parallel, the higher the heat flow through the temperature sources.

### 4.4. Macro-Level Simulation

Of particular interest are joint simulations of the whole chip, which include several subunits that receive heat from sources through heat exchangers and thermal interfaces, and loaded with a converter that converts the output voltage of the harvester to one of the standard voltages. To avoid overloading this article with multiple, even though interesting simulations, we offer the simplified macro simulation that will demonstrate the capabilities of the method. If desired, this simulation can be complicated by simply adding elements.

**Figure 14:**
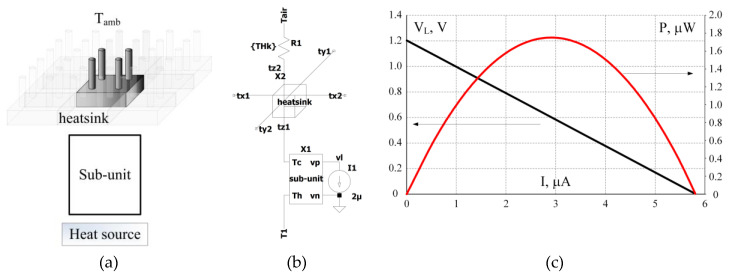
Fragment of the on-chip harvester including single sub-unit, heat source and fragment of a heatsink. (**a**) Block-diagram of the system, (**b**) equivalent circuit representation ar LTSpice. *T*_1_ = 35 °C is a heat source temperature, X1 is a hierarchical block of the circuit shown in Figure 13, X2 is a block of the heatsink base and the R1 is the thermal resistance of a part of the heatsink, (**c**) V-I and P-I of the circuit.

A fragment of a harvester on a chip is shown in Figure 14a. One of the nine subunits gets heat from the heat source and releases it into the surrounding air. Convection heat transfer is carried out using a heatsink. The thermal resistance between the substrate and the heat source is much less than the thermal resistance of the heatsink, so it can be neglected. A review of heatsinks shows that a heatsink with a base of 25 mm × 25 mm demonstrates thermal resistance of about 30 $K\cdot W^{-1}$ at zero airflow speed [32]. This means that the fragment of the heatsink (1/9 of its area) has approximately 9 times greater thermal resistance. Figure 14b shows the equivalent scheme in LTSpice and its load characteristics are shown in Figure 14c.

## 5. Validation of the Method and Comparison with a Proved Methodology

The method of spatial equivalent circuit modeling proposed in this study is derived from the finite difference method, in which the differential equations for an elemental volume of a three-dimensional grid are expressed in the form of equivalent electrical circuits. A similar technique is often used to solve thermal conductivity problems [21,24,25]. The well-known equivalent circuit widely used to solve thermal conductivity problems is expanded in this study. The equations of thermoelectric processes are added to the Fourier equation of the elemental volume and are also expressed as elements of an equivalent electrical circuit.

The objective of this section is to validate the legitimacy of using the proposed method in its expanded form. For this purpose, we chose a paper “Multiphysics simulation of thermoelectric systems-modeling of Peltier-cooling and thermoelectric generation” [33], in which the model of thermoelectric Peltier cooling and thermoelectric generation processes was simulated by means of the finite element method using COMSOL Multiphysics software (3.3a, COMSOL, Inc., Burlington, MA, USA). Simulations for steady-state processes under different boundary conditions and electric current values, as well as simulations of transients provided in the article, were reconstructed using the proposed method.

**Figure 15:**
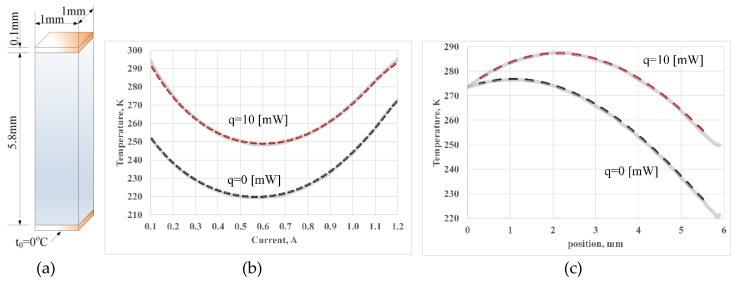
(**a**) A p-type thermoelectric element is contacted by copper electrodes, and steady-state simulation results: (**b**) the cold side temperature vs. current with and without thermal load, and (**c**) temperature distribution along the element calculated without and with a 10 mW heat load. The results obtained as a result of simulation by the proposed method are shown with dashed lines. The gray solid lines are taken for comparison from [33].

A simple cooler geometry consisted of one p-type (Bi_0.5_Sb_0.5_)_2_Te_3_ semiconductor element 1 mm × 1 mm × 5.8 mm in size (see Figure 15a). It was contacted by two copper electrodes 0.1 mm in thickness. The thermal, electrical, and thermoelectric properties of the material as function of temperature are tabulated in Table 4. The properties of copper are assumed temperature independent. The material properties of the copper, from [33], were as follows:$\;\alpha_{c}=6.5\;\left[\mu V\cdot K^{-1}\right],\;\sigma_{c}=5.9\times 10^{8},\;\left[S\cdot m^{-1}\right],\;and\;\lambda_{c}=350,\;\left[W\cdot m^{-1}\cdot K^{-1}\right]$.

### 5.1. Example 1: Steady-State Operation

The boundary conditions were set to 0 V and 0 °C at the base of the lower electrode. Adiabatic boundary conditions were taken on all other surfaces. At the top of the upper electrode, a current of 0.7 A was applied. The top side of the element under test was thermally isolated (thermally unloaded) or thermally loaded by 10 mW thermal load in rotation. The results of the simulation using the finite element method in COMSOL Multiphysics software presented in [33] are shown in the picture by solid gray lines. The graphics from [33] were digitized using Engauge digitizer software [34]. Figure 15b demonstrates the cold side temperatures versus current without and with 10 mW heat load, respectively.

In order to simulate the system using the method proposed in this study, the twelve hierarchical blocks of the elemental volumes that are shown in Figure 6 were used. (Ten blocks for the thermoelectric element model and two for the copper contacts). The dependence of the properties of the material on temperature was performed using the lookup tables in accordance with the values of Table 4. The blocks were connected in series along the *z*-axis both electrically and thermally. The electrical domain of the first block was connected to a 0 V electrical potential, and the thermal domain of the first block was connected to a source of the equivalent temperature of 273.15 K (0 °C). The electrical domain of the last block was connected to an electric current source and the thermal domain of the last block was loaded by the equivalent heat source.

Simulation results are shown by dashed lines in Figure 15b,c together with results obtained in [33]. Comparing the results, we can conclude that in the steady state, the proposed method gives practically identical results to the finite-element method.

### 5.2. Example 2. Transient Operation

**Figure 16:**
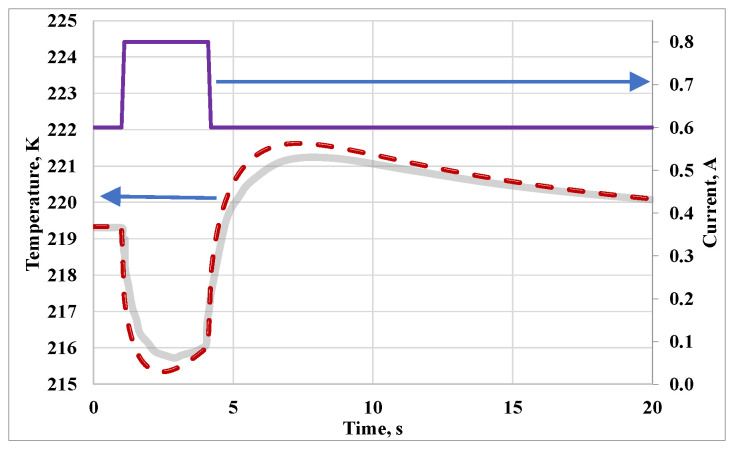
Transient calculation of Peltier-supercooling. A short current pulse (upper magenta line) generates temporarily lower cold side temperatures. The gray solid line demonstrates results of the finite element simulation from [33], the dashed line corresponds to a simulation by the proposed method.

Figure 16 demonstrates the reaction of the thermoelectric system to the current pulse in the time domain. The thermoelectric element described above is operated at 0.6 ampere with no heat load at steady-state temperatures. One second after the beginning of the calculation, the current is increased to 0.8 A linearly within 0.1 s. After 4 seconds the amperage decreases back to 0.6 A linearly within 0.1 s (see the upper curve in Figure 16). The solid gray line shows the transient cold side temperature of the element, with temperature-dependent material parameters simulated in COMSOL Multiphysics from [33]. The dashed line shows the results of transient simulation of the element by means of LTSpice circuit simulation software using the method proposed in the current study. A slight deviation of the obtained results from the results that we accepted as a reference is explained by the fact that the mesh we used in the equivalent circuit simulation has only 12 blocks. The number of nodes used to simulate finite element models in specialized software is usually significantly higher. Nevertheless, the results of calculations performed by two different methods have very close values and show the nature of the temperature change as a reaction to excitation in a similar way.

## 6. Discussion and Conclusions

The technique described above allows the simulation of thermal and thermoelectric processes together with electrical ones. The method is based on the creation of a two-domain (electrical and thermal) equivalent circuit of elemental volume with energy interrelations between the domains. The equations in both domains are solved for each of the three spatial axes; therefore, this method allows the simulation of processes for which the spatial arrangement matters.

The article is illustrated with examples of using the proposed method: 1. Modeling for three-dimensional time-domain analysis of the heat distribution in solids due to boundary conditions variation and internal heat generation. 2. Steady-state analysis of a planar on-chip thermoelectric couple, and a sub-unit including 1k couples. 3. Analysis of a thermoelectric sub-unit connected to heat/cold source through the heatsink. 4. The influence of the dependence of the thermodynamic and electrical parameters of the material on the local temperature in each of the mesh nodes is demonstrated in the example of the Thomson effect when the thermoelectric coefficient changes its value from node to node of the mesh, depending on the temperature of each of the nodes.

A hierarchical block, which includes a two-domain equivalent elemental volume scheme, is the basic element for creating models. Thermodynamic and electrical parameters of the material are the parameters of the corresponding hierarchical block. The more blocks involved in the simulation, the higher the spatial resolution, and therefore the accuracy.

Any software for electrical circuits simulation can provide the simulations using the proposed method. In this work, LTSpice, the SPICE-based simulator, was used to produce examples. The method allows for the use of all the main types of simulations used in engineering: steady-state (bias point, DC-sweep, etc.) analysis, time-domain (transient) analysis, as well as frequency domain (AC) analysis. Any external electrical components (such as load, PWM converter, energy storage, close-loop voltage regulator, etc.) can be connected to the ports of the electrical domain of the equivalent circuit and participate in the joint simulation.

A comparison of the results of simulations performed using the proposed method with similar simulations performed by other researchers using the finite element method shows a perfect match in a large number of examples and a relatively small difference in the rest of the examples.

One drawback of the method is the cumbersomeness of equivalent circuits, entailing high load on the computer when simulating complex systems with high resolution. The high resolution achievable in finite-element programs cannot be achieved. Yet, unlike the finite-element programs, this method allows for the assessment of the mutual influences of thermal, electrical, and thermoelectric processes during design. If the accuracy of the results is unsatisfactory, more accurate calculation methods should be used instead.

Currently, the proposed method is in the initial stage of its development and requires further research. In particular, a more advanced method of convective heat transfer modeling needs to be developed, as well as a mesh optimization technique.

## Figures and Tables

**Table 1:** Analogies between thermal and electrical processes.

Thermal Domain	Electrical Domain
T: temperature, [K]	φ: electrical potential, [V]
q: heat flow, [W]	i: electrical current, [A]
$\Theta_{x}=\frac{1}{k}\cdot \frac{\Delta x}{\Delta y\cdot \Delta z}\;$, $\Theta_{y}=\frac{1}{k}\cdot \frac{\Delta y}{\Delta x\cdot \Delta z}\;$, $\Theta_{z}=\frac{1}{k}\cdot \frac{\Delta z}{\Delta y\cdot \Delta x}\;$: Thermal resistances of the elemental volume in *x*, *y* and *z* directions, [K/W]	$R_{x}=\rho_{\Omega }\cdot \frac{\Delta x}{\Delta y\cdot \Delta z}$, $R_{y}=\rho_{\Omega }\cdot \frac{\Delta y}{\Delta x\cdot \Delta z}$, $R_{z}=\rho_{\Omega }\cdot \frac{\Delta z}{\Delta y\cdot \Delta x}$: Electrical resistances of the elemental volume in *x*, *y* and *z* directions, [Ω], where $\rho_{\Omega }$ is resistivity of the material
$C_{T}=c_{p}\cdot m$: thermal capacitance, [J/K]	C: electrical capacitance, [F]
$q_{j}=\Delta T_{j}/\Theta_{j}$: Fourier’s law along axis j	$i_{j}=\frac{{\Delta \varphi }_{j}}{R_{j}}$: Ohm’s law along axis j
$q_{C}=C_{T}\cdot \frac{dT_{C}}{dt}$: change of internal energy	$i_{C}=C\cdot \frac{dV_{C}}{dt}$: charge of a capacitor

**Table 2:** Parameters used in the simulation.

Type of Parameter	Parameter	Value	Units
Dimensions	*x*-direction, $L_{x}$	$100\times 10^{-3}$	m
	*y*-direction $L_{y}$	$9\times 10^{-3}$	m
	*z*-direction $L_{z}$	$3\times 10^{-3}$	m
Material	Density, ρ	$2.7\times 10^{3}$	$\mathrm{kg}\cdot m^{-3}$
	Specific heat, $c_{p}$	910	$J\cdot {\mathrm{kg}}^{-1}\cdot K^{-1}$
	Specific thermal conductivity, k at 20°C	200	$W\cdot m^{-1}\cdot K^{-1}$
	Thermal coefficient, $k_{0}$	$4\times 10^{-3}$	$K^{-1}$
	Resistivity, $\rho_{\Omega }$	$2.7\times 10^{-8}$	Ω·m
	Thermal coefficient, $\rho_{\Omega 0}$	$-0.2\times 10^{-3}$	$K^{-1}$
Parameters of simulation	Ambient air temperature, $T_{amb}$	25+273.15	K
	$T_{1}$	$T_{amb}+10$	K
	$T_{2}$	$T_{amb}+5$	K
	$T_{3}$	$T_{amb}$	K
	$V_{1}$ delayed by 0.5 s	$40\times 10^{-3}$	V

**Table 3:** Properties of the materials used in the thermoelectric couple under simulation.

Element	Description	Parameter
P-leg	Dimensions	$L_{x}=70\;\mu m,\;L_{y}=4\;\mu m,\;and\;L_{z}=0.2\;\mu m$
	Meshing along *x*, *y*, and *z* axes	6×1×1
	Thermodynamic properties	$\rho =2.2k,\left[\mathrm{kg}\cdot m^{-3}\right]\;,\;c=100,\left[J/\left(\mathrm{kg}\;\cdot K\right)\right]$
		$k^{*}=31.2,\;\left[W/\left(m\cdot K\right)\right],\;k_{0}=1\;m,\;\left[K^{-1}\right]$
	Electrical properties	$\rho_{\Omega }^{*}=1\;\mu ,\;\left[\Omega \cdot m\right],\;\rho_{\Omega 0}=5\;m,\;\left[K^{-1}\right]$
	Thermo-electric properties	$\alpha^{*}=103\;\mu ,\;\left[V\cdot K^{-1}\right],\;\alpha_{0}=3\;m$
N-leg	Dimensions	$L_{x}=70\;\mu m,\;L_{y}=4\;\mu m,\;and\;L_{z}=0.2\;\mu m$
	Meshing along *x*, *y*, and *z* axes	6×1×1
	Thermodynamic properties	$\rho =2.2k,\left[\mathrm{kg}\cdot m^{-3}\right]\;,\;c=100,\left[J/\left(\mathrm{kg}\;\cdot K\right)\right]$
		$k^{*}=31.5,\;\left[W/\left(m\cdot K\right)\right],\;k_{0}=1\;m,\;\left[K^{-1}\right]$
	Electrical properties	$\rho_{\Omega }^{*}=1.2\;\mu ,\;\left[\Omega \cdot m\right],\;\rho_{\Omega 0}=5\;m,\;\left[K^{-1}\right]$
	Thermo-electric properties	$\alpha^{*}=-57\;\mu ,\;\left[V\cdot K^{-1}\right],\;\alpha_{0}=3\;m$
Metal 1	Dimensions	$L_{x}=4\;\mu m,\;L_{y}=4\;\mu m,\;and\;L_{z}=4\;\mu m$
	Meshing along *x*, *y*, and *z* axes	1×1×1
	Thermodynamic properties	$\rho =2.7k,\left[\mathrm{kg}\cdot m^{-3}\right]\;,\;c=910,\left[J/\left(\mathrm{kg}\;\cdot K\right)\right]$
		$k^{*}=200,\;\left[W/\left(m\cdot K\right)\right],\;k_{0}=1\;m,\;\left[K^{-1}\right]$
Metal 2	Dimensions	$L_{x}=141\;\mu m,\;L_{y}=4\;\mu m,\;and\;L_{z}=2\;\mu m$
	Meshing along *x*, *y*, and *z* axes	5×1×1
	Thermodynamic properties	see metal 1
Substrate 1	Dimensions	$L_{x}=4\;\mu m,\;L_{y}=8\;\mu m,\;and\;L_{z}=4\;\mu m$
	Meshing along *x*, *y*, and *z* axes	1×1×1
	Thermodynamic properties	$\rho =2.33k,\left[\mathrm{kg}\cdot m^{-3}\right]\;,\;c=764,\left[J/\left(\mathrm{kg}\;\cdot K\right)\right]$
		$k^{*}=170,\;\left[W/\left(m\cdot K\right)\right],\;k_{0}=1\;m,\;\left[K^{-1}\right]$
Substrate 2	Dimensions	$L_{x}=141\;\mu m,\;L_{y}=8\;\mu m,\;and\;L_{z}=700\;\mu m$
	Meshing along *x*, *y*, and *z* axes	3×1×3
	Thermodynamic properties	See substrate 1

**Table 4:** Temperature dependent material properties of (Bi_0.5_Sb_0.5_)_2_Te_3_ from [33,35].

t,[°C]	T, [K]	$\alpha ,\;\left[\mu V\cdot K^{-1}\right]$	$\lambda ,\;\left[W\cdot m^{-1}\cdot K^{-1}\right]$	$\sigma ,\;\left[mS\cdot m^{-1}\right]$
−173.15	100	75	2.5	185
−123.15	150	125	2	142
−73.15	200	170	1.55	100
−23.15	250	200	1.35	72
26.85	300	218	1.28	60
76.85	350	225	1.35	55
126.85	400	218	1.75	70

## References

[1] Bennett G.L., Skrabek E.A. (1996). Power Performance of US Space Radioisotope Thermoelectric Generators. Proceedings of the Fifteenth International Conference on Thermoelectrics. Proceedings ICT’96.

[2] Ritz F., Peterson C.E. Multi-Mission Radioisotope Thermoelectric Generator (MMRTG) Program Overview. Proceedings of the 2004 IEEE Aerospace Conference Proceedings (IEEE Cat. No. 04TH8720).

[3] Amara-Madi S., Price C.A., Bensaoula A., Boukadoum M. Autonomous Sensor System for Deep-Sea Pipeline Monitoring. Proceedings of the 2013 IEEE 11th International New Circuits and Systems Conference (NEWCAS).

[4] Harrop P. Sea Buoys Adopt Energy Harvesting, Printed Electronics World. https://www.printedelectronicsworld.com/articles/1575/sea-buoys-adopt-energy-harvesting.

[5] Mead R.L. (1966). Radioisotope generators on the Earth In Power from Radioisotopes.

[6] Leonov V., Fiorini P., Sedky S., Torfs T., Van Hoof C. Thermoelectric MEMS Generators as a Power Supply for a Body Area Network. Proceedings of the 13th International Conference on Solid-State Sensors, Actuators and Microsystems, 2005. Digest of Technical Papers. TRANSDUCERS’05.

[7] Xie J., Lee C., Wang M.F., Liu Y., Feng H. (2009). Characterization of heavily doped polysilicon films for CMOS-MEMS thermoelectric power generators. J. Micromech. Microeng..

[8] Schaevitz S.B., Franz A.J., Jensen K.F., Schmidt M.A. (2001). A combustion-Based MEMS Thermoelectric Power Generator. Transducers’ 01 Eurosensors XV.

[9] Li Y., Buddharaju K., Singh N., Lo G.Q., Lee S.J. (2011). Chip-level thermoelectric power generators based on high-density silicon nanowire array prepared with top-down CMOS technology. IEEE Electron Device Lett..

[10] Wijngaards D.D., Kong S.H., Bartek M., Wolffenbuttel R.F. (2000). Design and fabrication of on-chip integrated polySiGe and polySi Peltier devices. Sens. Actuators A Phys..

[11] Boniche I., Masilamani S., Durscher R.J., Morgan B.C., Arnold D.P. (2009). Design of a miniaturized thermoelectric generator using micromachined silicon substrates. J. Electron. Mater..

[12] Liu K., Liu Y., Xu Z., Zhang Z., Yuan Z., Guo X., Jin Z., Tang X. (2017). Experimental prototype and simulation optimization of micro-radial milliwatt-power radioisotope thermoelectric generator. Appl. Therm. Eng..

[13] Sato N., Ishii H., Urano M., Sakata T., Terada J., Morimura H., Shigematsu S., Kudou K., Kamei T., Machida K. Novel MEMS Power Generator with Integrated Thermoelectric and Vibrational Devices. Proceedings of the 13th International Conference on Solid-State Sensors, Actuators and Microsystems, 2005. Digest of Technical Papers. TRANSDUCERS’05, IEEE.

[14] Goncalves L.M., Rocha J.G., Couto C., Alpuim P., Correia J.H. (2008). On-chip array of thermoelectric Peltier microcoolers. Sens. Actuators A Phys..

[15] Zhou H., Kropelnickib P., Tsai J.M., Leea C. (2014). Study of the Thermoelectric Properties of Heavily Doped Poly-Si in High Temperature. Procedia Eng..

[16] Yang M.Z., Wu C.C., Dai C.L., Tsai W.J. (2013). Energy harvesting thermoelectric generators manufactured using the complementary metal oxide semiconductor process. Sensors.

[17] Xie J., Lee C., Feng H. (2010). Design, fabrication, and characterization of CMOS MEMS-based thermoelectric power generators. J. Microelectromech. Syst..

[18] Lung C.L., Chien J.H., Chou Y.F., Kwai D.M., Chang S.C. (2012). Three-Dimensional Integrated Circuits Design for Thousand-Core Processors: From Aspect of Thermal Management. VLSI Des..

[19] Lineykin S., Ben-Yaakov S. (2007). User-friendly and intuitive graphical approach to the design of thermoelectric cooling systems. Int. J. Refrig..

[20] Ramírez-Laboreo E., Sagüés C., Llorente S. (2014). Thermal Modeling, Analysis and Control Using an Electrical Analogy. Proceedings of the 22nd Mediterranean Conference on Control and Automation.

[21] Lineykin S., Ben-Yaakov S. (2007). Modeling and analysis of thermoelectric modules. IEEE Trans. Ind. Appl..

[22] Shao Y., Li X.C., Mao J.F. (2012). An Electrothermal Model of Interconnects Based on a Transmissionline Network. Proceedings of the 2012 Asia Pacific Microwave Conference Proceedings.

[23] Buzilo R., Likhterov B., Giterman R., Levi I., Fish A., Belenky A. (2014). Approach to Integrated Energy Harvesting Voltage Source Based on Novel Active TEG Array System. Proceedings of the 2014 IEEE Faible Tension Faible Consommation.

[24] Salome P., Leroux C., Crevel P., Chante J.P. (1999). Investigations on the thermal behavior of interconnects under ESD transients using a simplified thermal RC network. Microelectron. Reliab..

[25] Holman J.P. (2009). Heat Transfer-Si Units-Sie.

[26] LeBlanc S., Yee S.K., Scullin M.L., Dames C., Goodson K.E. (2014). Material and manufacturing cost considerations for thermoelectrics. Renew. Sustain. Energy Rev..

[27] Zheng X.F., Liu C.X., Yan Y.Y., Wang Q. (2014). A review of thermoelectrics research–Recent developments and potentials for sustainable and renewable energy applications. Renew. Sustain. Energy Rev..

[28] Elsheikh M.H., Shnawah D.A., Sabri M.F., Said S.B., Hassan M.H., Bashir M.B., Mohamad M. (2014). A review on thermoelectric renewable energy: Principle parameters that affect their performance. Renew. Sustain. Energy Rev..

[29] Zhou M., Al-Furjan M.S., Zou J., Liu W. (2018). A review on heat and mechanical energy harvesting from human–Principles, prototypes and perspectives. Renew. Sustain. Energy Rev..

[30] LTSpice, SPICE Electronic Circuit Simulation Software, Analog Devices. https://www.analog.com/en/design-center/design-tools-and-calculators/ltspice-simulator.html.

[31] Carmo J.P., Gonçalves L.M., Correia J.H. (2009). Thermoelectric microconverter for energy harvesting systems. IEEE Trans. Ind. Electron..

[32] Cool Innovations Inc. http://www.coolinnovations.com/.

[33] Jaegle M. Multiphysics Simulation of Thermoelectric Systems-Modeling of Peltier-Cooling and Thermoelectric Generation. Proceedings of the COMSOL Conference 2008.

[34] Mitchell M., Muftakhidinov B., Winchen T. Engauge Digitizer Software. http://markummitchell.github.io/engauge-digitizer.

[35] Seifert W., Ueltzen M., Müller E. (2002). One-dimensional modelling of thermoelectric cooling. Phys. Status Solidi A.

